# *In Vitro* Antioxidant Activities and *in Vivo* Anti-Hypoxic Activity of the Edible Mushroom *Agaricus bisporus (Lange) Sing*. Chaidam

**DOI:** 10.3390/molecules201017775

**Published:** 2015-09-25

**Authors:** Hong-Ji Li, Hai-Yan Chen, Lin-Lin Fan, Zhi-Hua Jiao, Qi-He Chen, Ying-Chun Jiao

**Affiliations:** 1 Department of Food Science and Nutrition, Zhejiang University, Hangzhou 310058, China; E-Mails: 21313060@zju.edu.cn (H.-J.L.); 11213025@zju.edu.cn (L.-L.F.); jzhihua@zju.edu.cn (Z.-H.J.); 2 College of Chemistry and Biology Science, Lishui University, Lishui 323000, China; E-Mail: lschy1963@126.com; 3 Agriculture and Animal Husbandry College, Qinghai University, Xining 810016, China

**Keywords:** *Agaricus bisporus (Lange) Sing.* Chaidam, antioxidant activity, anti-hypoxic activity

## Abstract

With the rising awareness of a healthy lifestyle, natural functional foods have gained much interest as promising alternatives to synthetic functional drugs. Recently, wild *Agaricus bisporus (Lange) Sing.* Chaidam has been found and artificially cultivated for its thick fresh body and excellent taste, with its antioxidant and anti-hypoxic abilities unknown. In this work, the antioxidant potential of its methanolic, 55% ethanolic, aqueous extracts and crude polysaccharide was evaluated in different systems. The results showed that polysaccharide was the most effective in scavenging ability on 2,2-diphenyl-1-picrylhydrazyl (DPPH) and hydroxyl radicals, metal chelating activity and reducing power, with EC_50_ values of 0.02, 2.79, 1.29, and 1.82 mg/mL, respectively. Therefore, we further studied the anti-hypoxic activity of crude polysaccharide. The results turned out that polysaccharide (300 mg/kg) prolonged the survival time, decreased the blood urea nitrogen and lactic acid content as well as increased the liver glycogen significantly, compared with the blank control and the commercialized product Hongjingtian (*p* < 0.05). With such excellent activities, we purified the polysaccharide and analyzed its molecular weight (120 kDa) as well as monosaccharide components (glucose, fructose and mannose). This study indicated that wild *Agaricus bisporus (Lange) Sing.* Chaidam had strong potential to be exploited as an effective natural functional food to relieve oxidative and hypoxia stresses.

## 1. Introduction

Global industrialization and agricultural development lead to the release of various pollutants, including huge amounts of free radicals into the environment [1]. Oxidation is an essential biological process to many organisms for the production of energy. They can produce oxidative damage to DNA, protein and other macromolecules and have been postulated to be related to aging, various diseases, such as atherosclerosis, diabetes, cancer, cirrhosis [2], rheumatic arthritis [3] and food spoilage. Thus, developing natural antioxidant has attracted much attention.

Hypoxia is defined as a decrease in available oxygen reaching the tissues of the body, which can lead to body function impairment and may cause a variety of physiological abnormality [4]. To be specific, counter high altitude sickness or acute mountain sickness (AMS) is the most common symptom caused by hypoxia, which often occurs when people travel to high altitude. However, only acetazolamide is approved by the United States Food and Drug Administration to inhibit AMS, unfortunately it has side effects [5]. *Rhodiola algida*, also named Hongjingtian in Chinese, is the most famous medicine with anti-AMS effect and the first choice for people who travel to high altitude [6]. Therefore, developing natural antioxidant with anti-hypoxic activity is of great significance.

Edible mushrooms have been used in oriental culture as food-flavoring substances, nutritional food and also traditional Chinese medicines for many years because of their special fragrance and chemical composition. Additionally, mushrooms are believed to be harmless sources of natural antioxidants as they are rich in polysaccharides [7,8,9] and phenolic compounds [10,11], both of which are concerned with the antioxidant activity of fungi. Recently, a new kind of wild mushroom designated as *Agaricus bisporus (Lange) Sing.* Chaidam (*A. bisporus (Lange) Sing.* Chaidam), derived from Qinghai Plateau has been found and artificially cultivated due to its thick fresh body and excellent taste. To our best knowledge, the antioxidant and anti-hypoxia properties of this mushroom are still unknown. Therefore, the objective of this work was to examine and compare the antioxidant activity of polysaccharide and extracts from different solvents, as well as to evaluate the anti-hypoxic activity of the one with better antioxidant activity.

## 2. Results and Discussion

### 2.1. Antioxidant Activity

The antioxidant properties of the four extracts assayed herein are summarized in Table 1, and the results are normalized and expressed as EC_50_ values for comparison.

**Table 1:** EC_50_ values of four extracts from mushroom in antioxidant properties.

EC_50_ Value ^a^ (mg/mL)				
	Scavenging Ability on DPPH Radicals	Scavenging Ability on OH Radicals	Chelating Ability on Ferrous Ions	Reducing Power
MCE	0.29 ± 0.14 ^b^	6.10 ± 0.01 ^a^	4.34 ± 0.08 ^a^	2.90 ± 0.01 ^c^
ECE	0.97 ± 0.22 ^b^	2.90 ± 0.08 ^c^	1.49 ± 0.07 ^c^	4.92 ± 0.03 ^b^
ACE	2.95 ± 0.71 ^a^	4.26 ± 0.06 ^b^	1.77 ± 0.08 ^b^	16.42 ± 0.04 ^a^
Polysaccharide	0.02 ± 0.01 ^b^	2.79 ± 0.02 ^d^	1.29 ± 0.06 ^d^	1.82 ± 0.02 ^d^

^a^ EC_50_ value: 1,1-diphenyl-2-picrylhydrazyl (DPPH) or hydroxyl (OH) radicals were scavenged by 50%, ferrous ions were chelated by 50%, and the absorbance was 0.5 for reducing capability, respectively. Effectiveness of antioxidant properties inversely correlated with their EC_50_ values; Each value is expressed as mean ± SD (*n* = 3); means with different small letters within a column are significantly different (*p* < 0.05). MCE: methanolic crude extract; ECE: ethanolic crude extract; ACE: aqueous crude extract.

#### 2.1.1. DPPH Radical Scavenging Activity

DPPH• whose chemical property is very stable presents purple in methanol solution and its maximum absorption is at 517 nm. Antioxidants can pair with the single electron of DPPH• and make the absorption disappear gradually. At the same time, the discoloration increases with the increased number of acceptant electron. Hence, DPPH• is usually used as a substrate to evaluate antioxidative capability of antioxidants [12].

**Figure 1:**
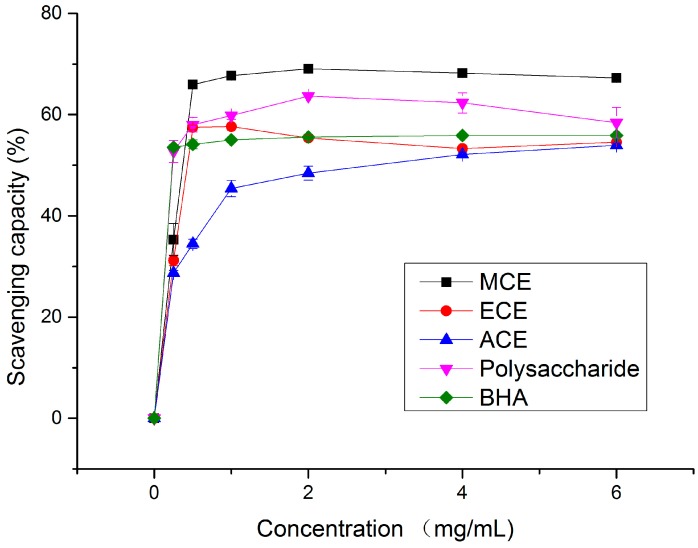
Free radical scavenging capacity of different concentrations of various extracts and BHA on 1,1-diphenyl-2-picrylhydrazyl radicals. MCE: methanolic crude extract; ECE: ethanolic crude extract; ACE: aqueous crude extract.

As can be seen in Figure 1, different mushroom extracts at various concentrations exhibited a dose dependent scavenging ability on DPPH• radicals. The scavenging effects of four extracts and the standard on DPPH• radical decreased in the order of MCE > Polysaccharide > butyl hydroxy anisd (BHA) > ECE > ACE, and, in the case of concentration of 6 mg/mL, the scavenging capacities came to 67.25%, 58.41%, 55.88%, 54.55% and 53.98%, respectively.

With regard to the EC_50_ values of the scavenging ability on DPPH radicals, the concentration of the polysaccharide was the lowest (0.02 ± 0.01 mg/mL) in comparison with MCE (0.29 ± 0.14 mg/mL), ECE (0.97 ± 0.22 mg/mL) and ACE (2.95 ± 0.71 mg/mL) (*p* < 0.05) (shown in Table 1) but higher than that of positive control BHA (0.01 ± 0.01 mg/mL). A large number of studies have reported that various mushroom extracts exhibit antioxidant activity, with the EC_50_ value as a measure. For instance, the methanolic extract of an edible mushroom *A. bisporus* was investigated with the EC_50_ value of 3.13 ± 0.09 mg/mL [13] and the aqueous extract of *C. purpurascens* was 0.37 ± 1.78 mg/mL [10]. In addition, Vaz *et al.* [14] have studied the EC_50_ values of ethanolic extract from four cultivated mushrooms, which ranged from 2.56 ± 0.31 mg/mL to 34.60 ± 0.44 mg/mL. Liu *et al.* [15] also revealed that the EC_50_ value of polysaccharide fraction of Jinqian mushroom was 1.47 mg/mL. Compared to the above results, four extracts from *A. bisporus* Chaidam all demonstrate a noticeable effect on scavenging DPPH radicals, acting possibly as primary antioxidants.

#### 2.1.2. Hydroxy Radical (•OH) Scavenging Activity

Hydroxy radical is the most active reactive oxygen species (ROS) known and it can lead to cell damage. Hence, scavenging hydroxy radical becomes one of the most effective way that organisms fight against diseases. Furthermore, Qi *et al.* [16] reported that hydroxyl radical scavenging activity was performed by indirect scavenging, but the inhibition of hydroxyl radical generation is by chelating ions such as Fe^2+^ and Cu^2+^.

**Figure 2:**
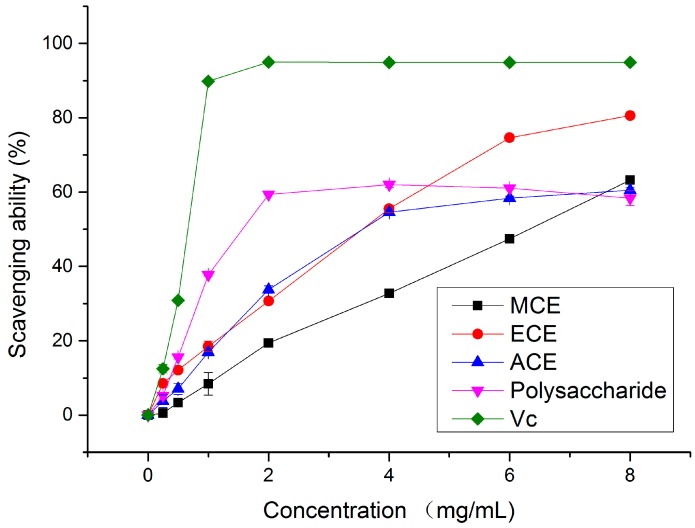
Free radical scavenging ability of different concentrations of various extracts and Vc on hydroxy radicals. MCE: methanolic crude extract; ECE: ethanolic crude extract; ACE: aqueous crude extract.

In this study, the MCE, ECE, ACE, polysaccharide and the standard were found to scavenge •OH directly to different extents. They exhibited excellent inhibition percentage of 0.56%–63.23%, 8.49%–80.57%, 3.77%–60.49%, 5.25%–61.97% and 12.46%–94.95% over a concentration range of 0.25–8 mg/mL, respectively (shown in Figure 2). In the case of a concentration of 8 mg/mL, the hydroxy scavenging activity of ECE was more effective than that of MCE, ACE and polysaccharide, but less effective than Vc used as the standard (94.95% at 2 mg/mL).

The EC_50_ values for hydroxy radical scavenging ability of different extracts and the standard were displayed in the following order: Vc (0.61 ± 0.01 mg/mL) > Polysaccharide (2.79 ± 0.02 mg/mL) > ECE (2.90 ± 0.08 mg/mL) > ACE (4.26 ± 0.06 mg/mL) > MCE (6.10 ± 0.01 mg/mL). It can be observed that the EC_50_ value of ECE gave a higher hydroxyl radical scavenging ability than that of *Agaricus bisporus* (5.23 mg/mL) [17] and *P. ostreatus* (8 mg/mL) [18]. The hot water extract showed higher scavenging ability compared to the species *P. ferulae* and *C. maxima* cap whose EC_50_ values were 11.7 ± 0.55 mg/mL and 55.1 ± 0.78 mg/mL, respectively [19]. However, the EC_50_ values of MCE and polysaccharide in this work were higher than the methanolic extract of *P. eous* (3.1 ± 0.08 mg/mL) [20] and the water-soluble polysaccharide of *Pleurotus tuber-regium* (Fr.) Sing. (0.14 mg/mL) reported earlier in this system [21]. All in all, the mushroom investigated in this study can be considered as a good scavenger of hydroxyl radical.

Depending on the aforementioned results, each extract can scavenge hydroxy radicals, which are known to be capable of abstracting hydrogen atoms from membrane and bringing about peroxidic reactions of lipids efficiently [22]. Based on this work, it was expected that the extracts would show antioxidant effects against lipid peroxidation on biomembranes. In the meantime, these extracts were also expected to scavenge the •OH and superoxide anions at the stage of initiation and termination of peroxy radicals [20]. Therefore, they can protect organism from the oxygen-derived free radicals.

#### 2.1.3. Metal Chelating Activity

**Figure 3:**
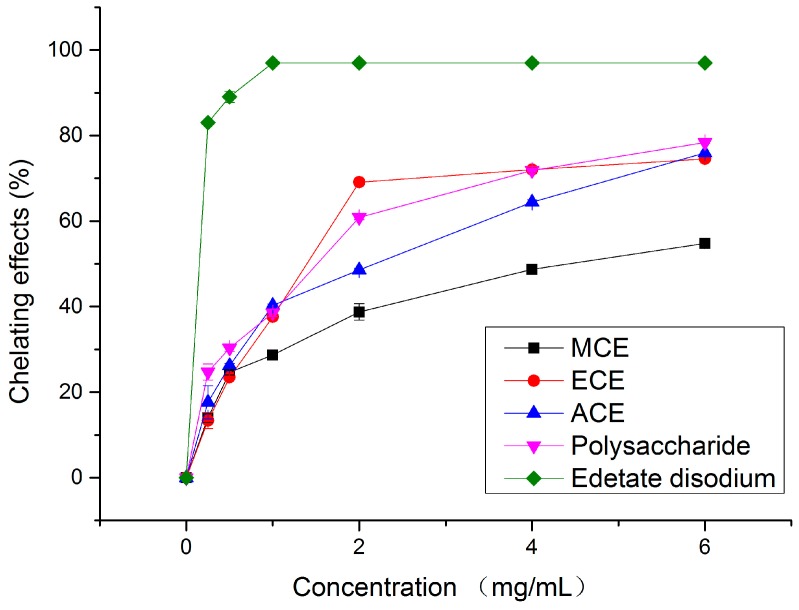
Metal chelating effects of different concentrations of four extracts and edetate disodium on ferrous ions. MCE: methanolic crude extract; ECE: ethanolic crude extract; ACE: aqueous crude extract.

Iron can stimulate lipid peroxidation by the Fenton reaction, and also lead to the formation of hydroxy radicals [12]. In addition, iron widely existing in food is commonly considered as an efficient pro-oxidant. Hence, metal chelating activity has been broadly accepted as a tool for estimating antioxidant ability of food-borne antioxidant. Ferrozine can quantitatively form complexes with Fe^2+^. In the presence of chelating agents, the complex formation is disrupted with the result that the red colour of the complex is decreased. Measurement of color reduction therefore allows estimation of the chelating activity of the coexisting chelator [23]. As indicated in Figure 3, the chelating activity of the various extracts on ferrous ions increased with their concentrations. The chelating percentage of MCE, ECE, ACE, polysaccharide (at 6 mg/mL) and the standard sample Edetate disodium were found to be 54.80%, 74.56%, 75.93%, 78.36% and 96.94%, respectively (Figure 3). The data in Table 1 revealed that the EC_50_ values of four extracts and the standard decreased in the order of MCE (4.34 ± 0.08 mg/mL) > ACE (1.77 ± 0.08 mg/mL) > ECE (1.49 ± 0.07 mg/mL) > Polysaccharide (1.29 ± 0.06 mg/mL) > Edetate disodium (0.02 ± 0.001 mg/mL) (*p* < 0.05). Up to now, abundant studies have reported the extracts from various fungi exhibited a potential metal iron-chelating activity, which is mostly measured by the EC_50_ value. For example, the chelating abilities of polysaccharide from *A. bisporus*, *A. brasiliensis*, *G. lucidum* and *P. linteus* have been investigated with EC_50_ values ranging from 0.59 ± 0.01 mg/mL to 7.80 ± 0.21 mg/mL [24]. When it refers to ethanolic extract, Guo *et al.* [25] revealed that the EC_50_ value of *Tuber indicum* was 0.98 ± 0.003 mg/mL. Besides, the methanolic extracts from 16 species of mushrooms were tested with a good metal chelating activity by Witkowska *et al.* [26], and their EC_50_ values were higher than the results in this study. Thus, we can conclude that the formation of Fe^2+^-ferrozine complex can be prevented by various extracts of this mushroom. Moreover, extracts of the wild edible mushroom demonstrated a marked capacity for iron binding, indicating that their function as peroxidation protector may be related to its iron binding capacity [27].

#### 2.1.4. Reducing Power

**Figure 4:**
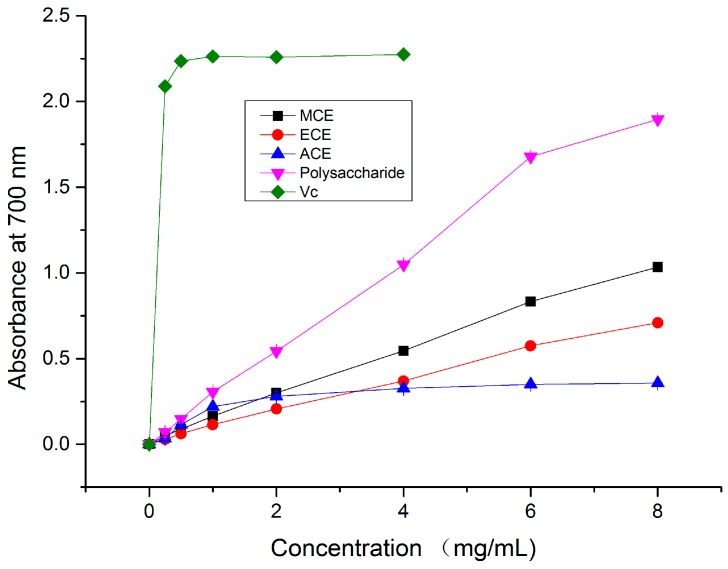
Comparison of reducing power of different extracts and Vc by spectrophotometric detection of the Fe^3+^–Fe^2+^ transformation at 700 nm. MCE: methanolic crude extract; ECE: ethanolic crude extract; ACE: aqueous crude extract.

The antioxidant can reduce the process of ferric (Fe^3+^) turning into ferrous (Fe^2+^) by donating an electron. The procedure can be measured spectrophotometrically at 700 nm. Therefore, this kind of hydrogen-donating ability may serve as a significant indicator of corresponding antioxidant activity. The crude polysaccharide extract showed an excellent reducing power of 1.90 ± 0.002 at 8 mg/mL, followed by MCE (1.03 ± 0.001), ECE (0.71 ± 0.006) and ACE (0.36 ± 0.003). However, the reducing power of each extract was inferior to that of Vc (2.27 at 4 mg/mL) (Figure 4). As shown in Table 1, the EC_50_ value of polysaccharide (1.82 ± 0.02 mg/mL) is significantly less than that of MCE (2.90 ± 0.01 mg/mL), ECE (4.92 ± 0.03 mg/mL) and ACE (16.42 ± 0.04 mg/mL) (*p* < 0.05). It was worth noting that the EC_50_ value of aqueous extract was extremely high. Presumably, the active ingredients of the mushroom that could react with free radicals to stabilize and block radical chain reactions maybe extracted less by water. In addition, the studied ECE of *A. bisporus (Lange) Sing.* Chaidam gave a higher reducing power than the methanolic extract of *Cordyceps militaris* (L.) (5.55 ± 0.03 mg/mL) [28] but a lower one than the ethanolic extract of *Clitocybe odora* (3.63 ± 0.14 mg/mL) [14]. In conclusion, the three extracts except ACE all showed an excellent reducing power, implying its tremendous possibility to be exploited as natural antioxidant.

Therefore, polysaccharide extracted from *A. bisporus (Lange) Sing.* Chaidam showed more antioxidant activity than extracts from different solvents. According to the aforementioned result, we chose polysaccharide to observe its anti-hypoxic activity.

### 2.2. Anti-Hypoxic Activity

#### 2.2.1. Effect on the Survival Time Test

**Figure 5:**
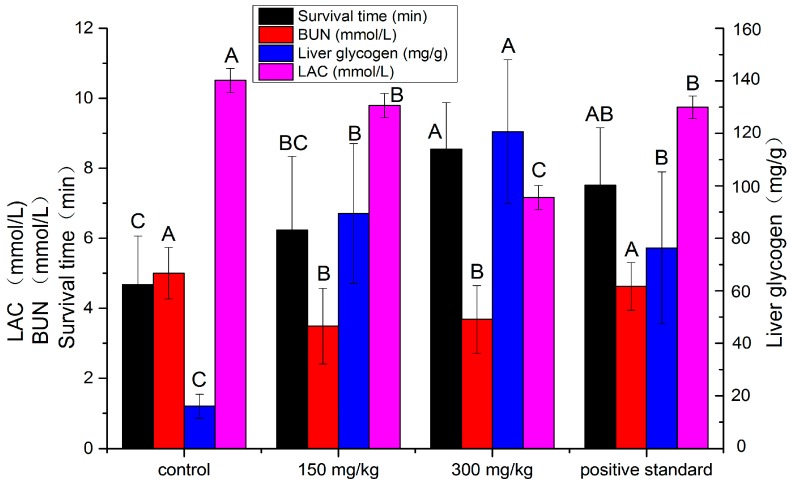
Effects of polysaccharide on survival time, blood urea nitrogen (BUN), liver glycogen and lactic acid (LAC) on mice. Means with different letters indicate significant differences at *p* < 0.05 according to DMRT. BUN: blood urea nitrogen; LAC: lactic acid.

As shown in Figure 5, the treatment with the polysaccharide (300 mg/kg) and Hongjingtian (280 mg/kg) prolonged significantly the survival time of oxygen deprivation, compared to the control group (*p* < 0.05). On the other hand, there was no significant difference between polysaccharide (300 mg/kg) and Hongjingtian (280 mg/kg) (*p* > 0.05). The data exhibited that the polysaccharide had a dose-dependent effect on increasing the survival time in mice exposed to hypoxia and showed as active as the standard at high dose.

#### 2.2.2. Effect on the Blood Urea Nitrogen Test

To provide energy, protein decomposition increases in organism under hypoxic condition, resulting in the increase of urea. The increment of blood urea nitrogen implies the level of energy supplying deriving from protein. Compared with control group, the polysaccharide (150 mg/kg and 300 mg/kg) showed a significant effect on decreasing the BUN test of mice (*p* < 0.05) and the Hongjiantian (280 mg/kg) decreased the BUN but showed no significant difference (*p* > 0.05) (Figure 5). The group of the dose (150 mg/kg and 300 mg/kg) exhibited the powerful activity in decreasing the BUN, which meant that the extract can ease the hypoxic stress.

#### 2.2.3. Effect on the Liver Glycogen Test

Energy for exercise is derived initially from the breakdown of glycogen and, later, from circulating glucose released by the liver [29]. Thus, liver glycogen is a sensitive parameter for our study. The more liver glycogen produced, the better anti-hypoxic activity showed. In the liver glycogen test (Figure 5), the treatment with the polysaccharide (300 mg/kg) showed the most significantly increase effect on the liver glycogen test than the other groups (*p* < 0.05). There was no significant difference between lower polysaccharide treatment (150 mg/kg) and the commercialized standard (280 mg/kg), but both of which increased significantly more than the control (*p* < 0.05). Therefore, the polysaccharide showed excellent activity in increasing the liver glycogen than the commercialized standard and the control.

#### 2.2.4. Effect on the Lactic Acid Test

The lactic acid content in blood is an indicator of anaerobic respiration, that is, a higher LAC content represents lower anti-hypoxic activity [30]. The control group showed highest LAC content of the four groups (*p* < 0.05). The lower dose polysaccharide and Hongjingtian group exhibited more anti-hypoxic activity than the control group (*p* < 0.05), but less than the higher dose polysaccharide group (*p* < 0.05). We can make a conclusion that the polysaccharide showed excellent anti-hypoxic activity.

### 2.3. Molecular Weight and Monosaccharide Compositions

The polysaccharide was obtained from the fruit body of *A. bisporus (Lange) Sing.* Chaidam by the method of water-extraction, ethanol-precipitation and a series of purification. The GPC analysis showed that the average molecular weight (Mw) of our sample was approximately 120 kDa. Monosaccharide compositions of our sample were glucose, fructose and mannose determined by the trifluoroacetic acid hydrolysis and GC-MS analysis. As for the specific molar ratio of these monosaccharides and the clear structure of our sample are ongoing. With such excellent activities and basic information known, it can be further exploited.

## 3. Experimental Section

### 3.1. Sample Preparation

The species of fresh mushroom (*A. bisporus* Chaidam) was obtained from Qinghai Province, China. We identified it through morphological and molecular strategies with ITS1 and ITS4. It turned out to be a kind of *Agaricus bisporus.* Because of its origin, we first named it *Agaricus bisporus (Lange) Sing.* Chaidam. Fruit bodies of this mushroom were cleaned to remove any residual compost, cut into pieces and then freeze-dried. All dried mushrooms were then ground by a mill into powder.

### 3.2. Extraction

Three different solvents (55% ethyl alcohol, methyl alcohol and water) were designed to fractionate the soluble compounds from the mushroom in different polarity. For ethanolic and methanolic extractions, a subsample (5 g) was extracted by using a graham condenser at 60 °C for 2 h with 40 mL of ethanol/methanol under reflux condition and filtered. The residue was then extracted with 40 mL ethanol/methanol as described above. The combined ethanolic and methanolic extracts were then concentrated under reduced pressure at 40 °C to dryness.

For aqueous extraction, a subsample (5 g) was extracted by stirring with boiling water (50 mL) at 100 °C for 10 min (800 r/min) and filtered. The residue was then extracted with two additional 50 mL portions of boiling water as described above. The combined hot water extracts were then freeze-dried.

For the preparation of crude polysaccharide, a subsample (5 g) was extracted using a graham condenser at 95 °C for 2 h with 75 mL of distilled water under reflux condition and filtered. The residue was then extracted with 75 mL aqueous as described above. The combined water extracts were then concentrated under reduced pressure at 45 °C to extract cream, deproteinized with Sevag reagent and precipitated with 95% ethyl alcohol (4:1, *v*/*v*). After centrifugation at 3000 r/min for 10 min, we got the crude polysaccharide by drying it at 60 °C.

All the four dried extracts including MCE (methanolic crude extract), ECE (ethanolic crude extract), ACE (aqueous crude extract) and polysaccharide were redissolved in the corresponding solvent to a concentration of 20 mg/kg and stored at 4 °C for further use.

### 3.3. Antioxidant Activity Assays

Scavenging activities on 1, 1-diphenyl-2-picrylhydrazyl (DPPH) radicals [31] and hydroxyl radicals [25], chelating metal ions activity [24] and reducing power [19] were assayed to determine the antioxidant activity of the four extracts thoroughly.

### 3.4. Animal

Male Qinghai mice (200~250 g) were purchased from the Center of Experimental Animals, Lanzhou University. They were housed at 21 ± 1 °C under a 12 h light/dark cycle and had free access to standard pellet diet (Purina chow) and tap water. Each mouse was used only once in the experiment. All animal treatments were strictly in accordance with international ethical guidelines and the National Institutes of Health Guide concerning the Care and Use of Laboratory Animals. The experiments were carried out with the approval of the Committee of Experimental Animal Administration of the Lanzhou University.

### 3.5. Pharmacological Studies

#### 3.5.1. Survival Time Test

After 3 days of adaptive feeding, mice were randomly divided into 4 groups with ten animals in each group, namely, normal control group, hypoxia model groups underlying different doses and Hongjingtian group as a positive control. The polysaccharide solutions (150 mg/kg/day and 300 mg/kg/day) and Hongjingtian (280 mg/kg/day) were administered intragastrically to the mice for a week. Then, we put the mice into hypobaric cabin (Cabin pressure: 50–58 kPa, Oxygen partial pressure: 11.0–11.3 kPa) for 72 h, feeding in the interval of 24 h. Sixty minutes after last treatment, each mouse was put into a 250 mL airtight container with 5 g medical soda lime. The survival time of oxygen deprivation from the time the bottle was sealed to when the mouse stopped breathing was recorded.

#### 3.5.2. Blood Urea Nitrogen (BUN)

About 0.5 mL of blood samples were withdrawn from the fossa orbitalis of the mice as soon as they stopped breathing. The blood was citrate-stabilized and centrifuged at 2000 rpm for 5 min. The blood plasma was separated and used to determine the content of BUN following the manufacturer’s instructions (Urea assay kit, Nanjing Jiancheng Bioengineering Institute, Nanjing, China).

#### 3.5.3. Liver Glycogen

Liver glycogen was measured according to the manufacturer’s instructions (Liver/Muscle glycogen assay kit, Nanjing Jiancheng Bioengineering Institute). The liver tissue were initially rinsed with saline, blotted by filter paper and hydrolyzed in alkali solution in boiling water for 20 min. A 1% solution of liver tissue were prepared and further mixed into glycogen test fluid. The absorbance of the products was measured at 620 nm and the results were expressed as mg/g.

#### 3.5.4. Lactic Acid (LAC)

About 0.5 mL of blood samples were withdrawn from the fossa orbitalis of the mice as soon as they stopped breathing. The blood was citrate-stabilized and centrifuged at 2000 rpm for 5 min. The blood plasma was separated and used to determine the content of LAC following the manufacturer’s instructions (Lactic acid assay kit, Nanjing Jiancheng Bioengineering Institute).

### 3.6. Isolation, Purification and Identification of Polysaccharide

The crude polysaccharide was dissolved in distilled water, centrifuged and injected to a DEAE-52 cellulose column with a gradient elution by NaCl solution at a flow rate of 1.0 mL/min. Fractions were collected by a fraction collector and further purified with a Sephadex G-100 column with distilled water as the eluents at a flow rate of 0.3 mL/min. After that, the carbohydrate contents of the sample were quantified by the phenol-sulfuric acid method [32]. Collect, dialyze and freeze-dry the peak with the highest polysaccharide content to get our sample.

The molecular weight of the polysaccharide was evaluated and determined by gel permeation chromatography (GPC), in a combination high-performance liquid chromatography apparatus [33].

The polysaccharide sample (20 mg) was hydrolyzed by trifluoroacetic acid (TFA) (2 M, 4 mL) at 120 °C for 6 h into monosaccharide, which was further silylated for GC analysis [34].

### 3.7. Statistical Analysis

All the experimental results were the mean (±standard deviation) of three parallel measurements for antioxidant activity and eight parallel measurements for anti-hypoxic activity. The data were analyzed using SPSS software. Analysis of variance and Duncan’s multiple range test (DMRT) were used to determine the least significance difference (LSD) amongst means at the level of 0.05.

## 4. Conclusions

In summary, this study revealed that MCE, ECE, ACE and polysaccharide extracted from wild *A. bisporus (Lange) Sing.* Chaidam had high antioxidant activity in all assays with lower EC_50_ values. To be specific, the polysaccharide showed more antioxidant activity than those extracts from different solvents. Therefore, we further investigated the anti-hypoxic activity of polysaccharide (150 mg/kg and 300 mg/kg), compared with the blank control and the commercialized product Hongjingtian (280 mg/kg) as the positive control. The results turned out that the polysaccharide possessed powerful anti-hypoxic activity, even better than the standard on BUN, liver glycogen and LAC tests at the concentration of 300 mg/kg. The average molecular weight of our purified sample was approximately 120 kDa and its monosaccharide compositions were glucose, fructose and mannose. The information obtained from the current research would be helpful for promotion of the cultivation and consumption of the edible *A. bisporus* Chaidam, which has the potential to be utilized as natural resources for functional and flavoring food, cosmetics and medical applications.

## Abbreviations

DPPH, 2,2-diphenyl-1-picrylhydrazyl; BHA, butyl hydroxy anisd; MCE, methanolic crude extract; ECE, ethanolic crude extracts; ACE, aqueous crude extract; LAC, lactic acid; BUN, blood urea nitrogen; *A. bisporus (Lange) Sing.* Chaidam, *Agaricus bisporus (Lange) Sing.* Chaidam; GPC, Gel Permeation Chromatography.
